# Unusual Presentation of Gastric Outlet Obstruction Due to Breast Cancer Metastasis: A Case Report

**DOI:** 10.7759/cureus.4533

**Published:** 2019-04-24

**Authors:** Nabeeha Mohy-ud-din, Bonnie Patek, Manish Dhawan

**Affiliations:** 1 Internal Medicine, Allegheny Health Network, Pittsburgh, USA; 2 Gastroenterology and Hepatology, Allegheny Health Network, Pittsburgh, USA

**Keywords:** gastric outlet obstruction, breast adenocarcinoma, endoscopic ultrasound, upper gastrointestinal endoscopy

## Abstract

Gastric outlet obstruction can be caused by various pathologies, including peptic ulcer disease, gastric polyps, and malignancies. The incidence rate of breast cancer metastasis to the stomach is only 0.3%. We describe a rare case of an 83-year-old female with a remote history of breast cancer who presented with symptoms of nausea and vomiting. She underwent an upper endoscopy, and biopsies revealed chronic gastritis. However, when she presented for the second time with similar symptoms, she underwent endoscopic ultrasound (EUS)-guided biopsies, which clinched the diagnosis of breast cancer metastasis causing gastric outlet obstruction. This case describes the importance of keeping a wide differential diagnosis for the causes of gastric outlet obstruction and the significance of deeper EUS-guided biopsies if initial endoscopic biopsies are inconclusive.

## Introduction

Gastric outlet obstruction (GOO) has multiple etiologies, including peptic ulcer disease, malignancies, and gastric polyps. With the identification of Helicobacter pylori and the use of proton pump inhibitors, the etiology of gastric outlet obstructions has changed over the years with malignancy now being the most common cause [1]. The most common malignancy to cause GOO is primary gastric adenocarcinoma, followed by carcinoma of the pancreas and gallbladder resulting in extrinsic compression of the duodenum or the stomach [2]. The estimated incidence rate of breast cancer metastasis to the stomach is approximately 0.3% with lobular breast adenocarcinoma more frequently observed than ductal breast adenocarcinoma in unusual sites such as the stomach [3]. The diagnosis of gastrointestinal metastasis is challenging. Although endoscopic mucosal biopsies can confirm the diagnosis, patients may require deeper biopsies due to tumor infiltration of layers deeper to the mucosa [4]. We report an unusual case of gastric outlet obstruction secondary to metastatic lobular breast carcinoma.

## Case presentation

An 83-year-old female presented to our emergency department with complaints of nausea and vomiting for four days. She had been diagnosed with right-sided, multicentric, infiltrating lobular carcinoma of the breast (Stage 1A, estrogen receptor positive (ER+), progesterone receptor positive (PR+), human epidermal growth factor receptor 2 negative (HER2-) 10 years ago. She had undergone a right mastectomy, and her sentinel lymph nodes, which were sampled during surgery, were negative for metastases. Previously, she had been treated with adjuvant anastrozole for five years, and yearly mammograms had been negative for recurrence.

**Figure 1:**
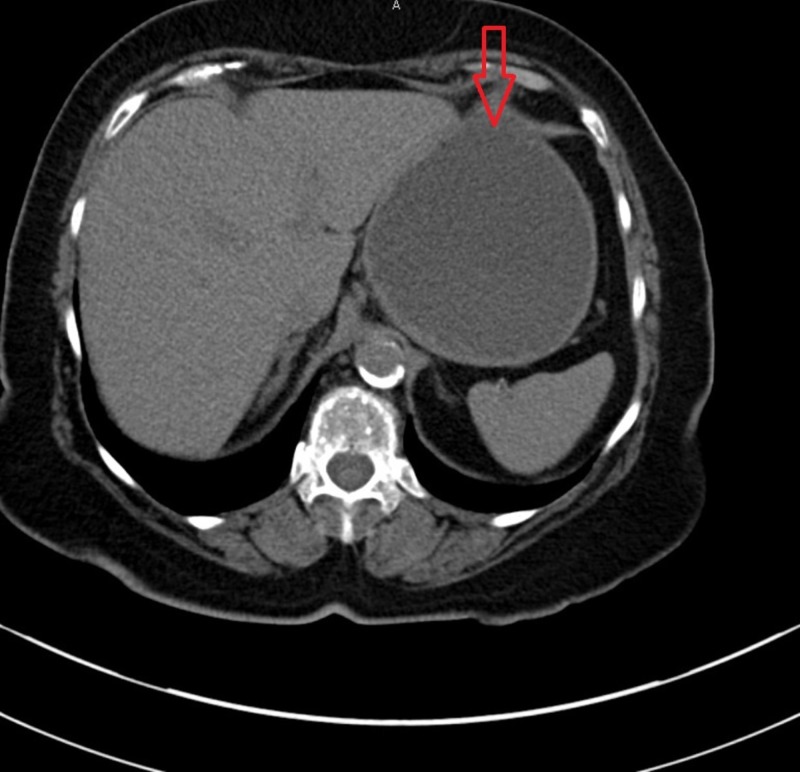
A computed tomography (CT) scan of the abdomen and pelvis showing a dilated stomach with gastric outlet obstruction.

One year prior to this presentation, she was evaluated at our hospital for similar complaints of nausea and vomiting. A computed tomography (CT) scan of her abdomen and pelvis on admission revealed a mass-like thickening of the gastric antrum and distension of the proximal stomach, as illustrated in Figure 1.

An upper endoscopy (EGD) was performed, which revealed esophagitis and gastric stenosis. This was dilated using a through-the-scope controlled radial expansion (CRE) balloon (Boston Scientific Inc., MA, US) to a maximum balloon size of 12 mm without fluoroscopic guidance. Biopsy of the gastric stenosis revealed gastric mucosa of antral type with minimal chronic inactive gastritis. No morphologic evidence of a Helicobacter pylori infection was detected. The patients’ symptoms of nausea and vomiting improved following balloon dilation. She was subsequently discharged on a daily proton pump inhibitor.

**Figure 2:**
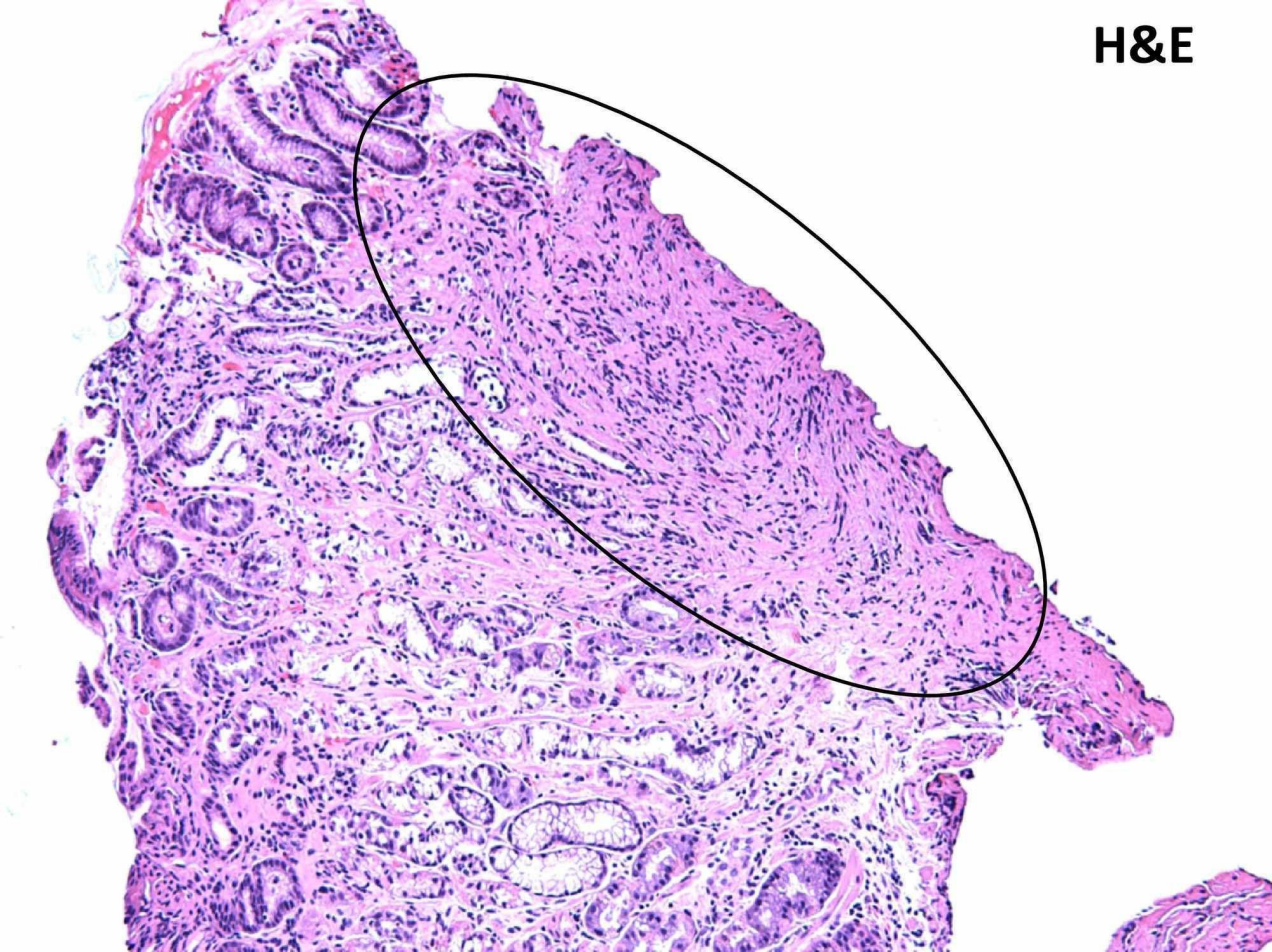
Histological findings from the gastric biopsy specimen: Encircled area with H&E staining revealing infiltration by poorly differentiated adenocarcinoma cells. H&E: Hematoxylin and eosin

The patient underwent endoscopic ultrasound (EUS) 12 weeks later. Gastric stenosis was found at the pylorus and duodenal bulb, which was dilated again with a CRE balloon to a maximum dilation of 13.5 mm. Diffuse wall thickening of the antrum of the stomach was visualized endosonographically. The gastric wall measured up to 11 mm in thickness. Thickening within the deep mucosa, submucosa, and muscularis propria was noted. EUS-guided biopsies were taken, which revealed invasive poorly differentiated metastatic breast adenocarcinoma, as shown in Figure 2.

**Figure 3:**
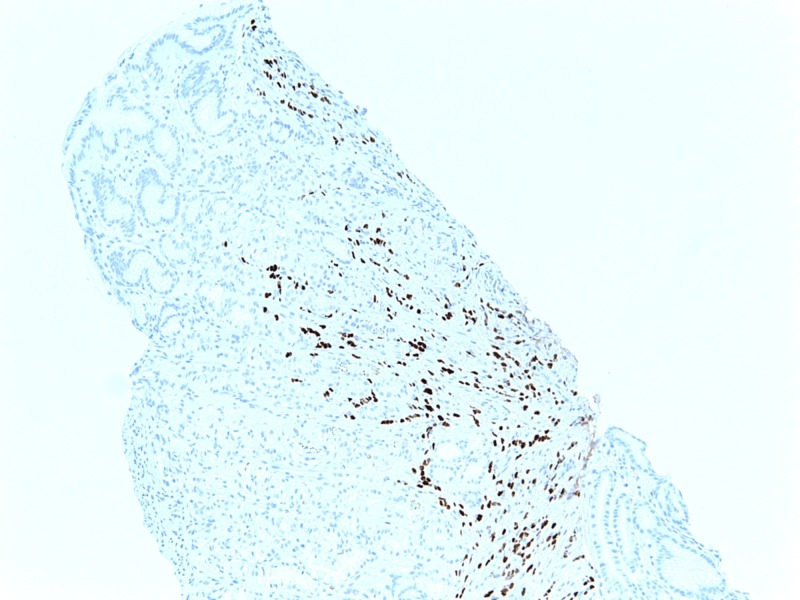
Immunohistochemical examination of the biopsy of the adenocarcinoma of the lobular breast, demonstrating ER positivity. ER: Estrogen receptor

**Figure 4:**
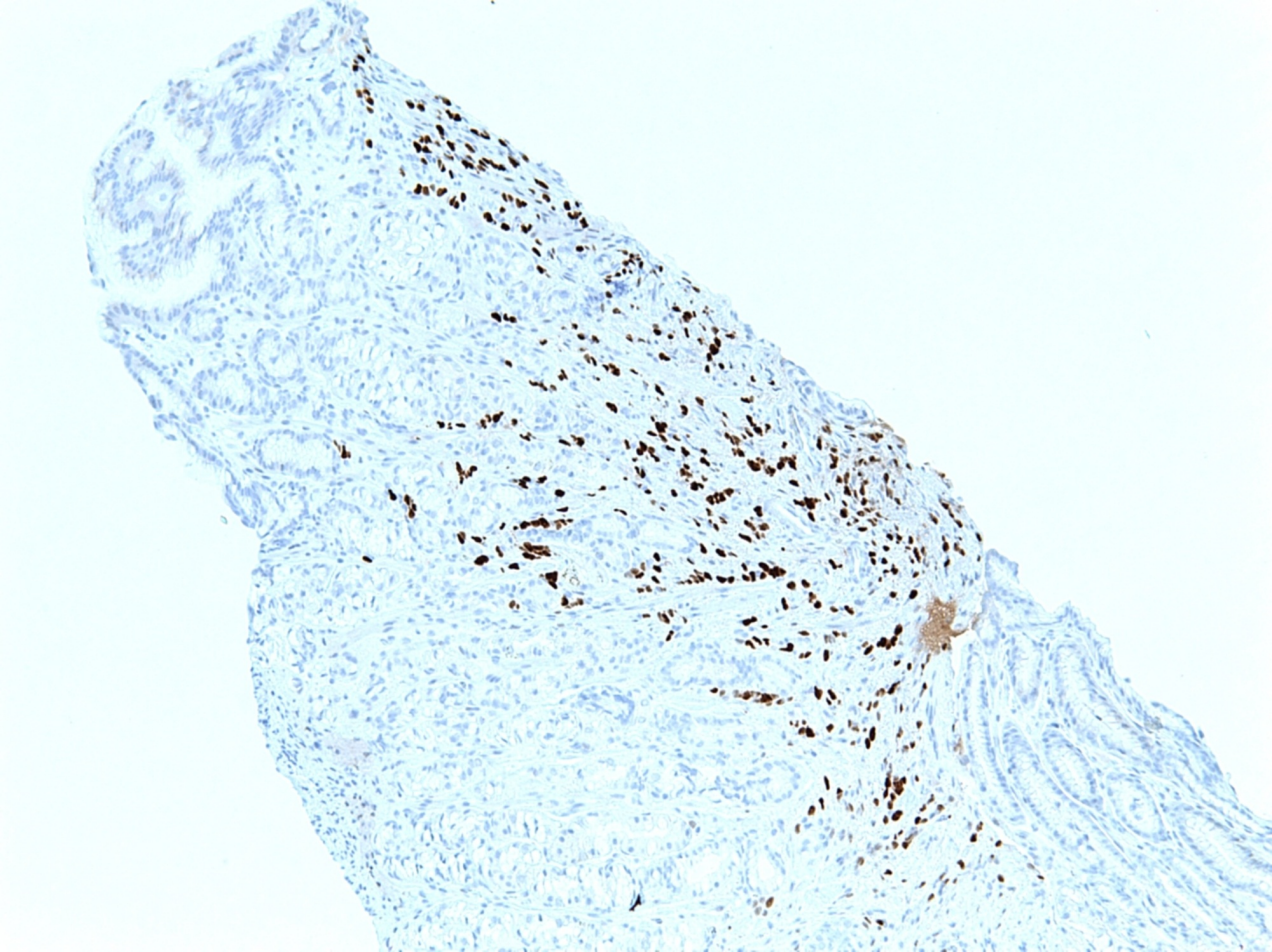
Immunohistochemical examination of the biopsy of the adenocarcinoma of the lobular breast, demonstrating PR positivity. PR: Progesterone receptor

Tumor immunohistochemistry and morphology revealed ER+, PR+, and HER2- negative lobular breast adenocarcinoma as demonstrated in Figure 3 and Figure 4.

## Discussion

This case highlights the importance of keeping a broad differential diagnosis for cases presenting with gastric outlet obstruction, especially in patients with a history of cancer. A diagnosis of breast cancer metastases to the gastrointestinal tract is often challenging because of its low incidence. Distinguishing between metastatic breast carcinoma and primary gastric adenocarcinoma cannot always be done using histological examination alone, and superficial biopsies may not be sufficient. Invasive lobular breast carcinoma and primary gastric cancer can have similar histopathology, as both can show signet ring cells. Immunohistochemistry is needed to make a distinction between the two, based on staining for estrogen and progesterone receptors [5]. As in our patient, the initial endoscopic mucosal biopsy reported chronic inactive gastritis, whereas a EUS-guided biopsy then revealed metastasis from breast cancer.

An important differential diagnosis for patients with concurrent gastric and breast adenocarcinoma includes hereditary diffuse gastric cancer (HDGC), which is an inherited cancer syndrome associated with an increased risk for primary gastric cancer and lobular breast carcinoma and is characterized by a poor prognosis. CDH1 gene mutations are frequently associated with HDGC and patients with this syndrome may benefit from genetic counseling.

Ulmer et al. described a similar case to ours where previous gastric mucosal biopsies had been negative; however, a EUS-guided fine needle aspiration clinched the final diagnosis of metastatic lobular breast carcinoma causing gastric outlet obstruction [6]. Of note, the EUS-guided biopsy in our case was a mucosal biopsy and not a fine needle aspiration (FNA) biopsy. It is vital to distinguish between breast cancer metastasis to the stomach and primary gastric cancer because treatment for the metastatic tumor usually involves systemic chemotherapy rather than a local treatment for gastric lesions [7]. Taal et al. reported that in a large series of 2000 endoscopic examinations, biopsies were negative in 20% patients with gastric metastases, which supports the notion that the differential diagnosis of the disease is an important strategy [8].

## Conclusions

Although case reports of breast cancer metastasis causing GOO do exist in the literature, our case emphasizes the need to be diligent and thorough with a low threshold for EUS-guided biopsies if the initial endoscopic biopsy is inconclusive. This is critical in order to avoid delay in care for a patient with metastatic carcinoma.
